# Editorial: A multi-talented butterfly: the role of the cerebellum in social cognition, emotion, and language

**DOI:** 10.3389/fnhum.2023.1322977

**Published:** 2023-11-03

**Authors:** Qianying Ma, Kris Baetens, Cleo L. Crunelle, Elien Heleven

**Affiliations:** ^1^Language Pathology and Brain Science MEG Lab, School of Communication Sciences, Beijing Language and Culture University, Beijing, China; ^2^Brain, Body and Cognition, Center for Neuroscience, Vrije Universiteit Brussel, Brussel, Belgium; ^3^Department of Psychiatry, Vrije Universiteit Brussel, University Hospital Brussels (UZ Brussel), Brussel, Belgium

**Keywords:** cerebellum, social cognition, language, emotions, connectivity, psychiatry

In our daily life, understanding what others think, inferring their emotions and communicating with each other can facilitate smooth social interactions. Over the last decades, researchers have been devoted to exploring how the cerebrum processes information in these social interactions. The human cerebellum has a surface that is about 80% of that of the cerebral cortex and has about four times as many neurons (Lent et al., 2012; Sereno et al., 2020). With this in mind, it is surprising that neuroscientists have only relatively recently become interested in cerebellar involvement in social, emotional and linguistic processes (See meta-analyses: Buckner et al., 2011; Van Overwalle et al., 2020a,b, 2023).

Going beyond the traditional understanding of the cerebellum as a motor controller (Manto et al., 2012), there is a growing awareness that the cerebellum also plays a critical role in non-motor domains; from basic cognitive functions to complex social functions. Multiple meta-analyses conducted during the last decade have shown that the cerebellum, especially the posterior cerebellum, is robustly and consistently activated during non-motor tasks (Van Overwalle et al., 2014, 2023; Guell et al., 2018; King et al., 2019).

A recent large-scale meta-analysis examined over 1000 fMRI studies encompassing more than 44,500 participants regarding task-based functional organization within the cerebellum (Van Overwalle et al., 2023). This parcellation suggested that there are 10 functional clusters associated to seven networks, similar to what was proposed by Buckner et al. (2011). As shown in Figure 1, the task-based parcellation demonstrated the involvement of the cerebellum, especially of the posterior cerebellar Crus I and II and the inferior posterior cerebellar IX, in social mentalizing, language and affective task contexts.

**Figure 1:**
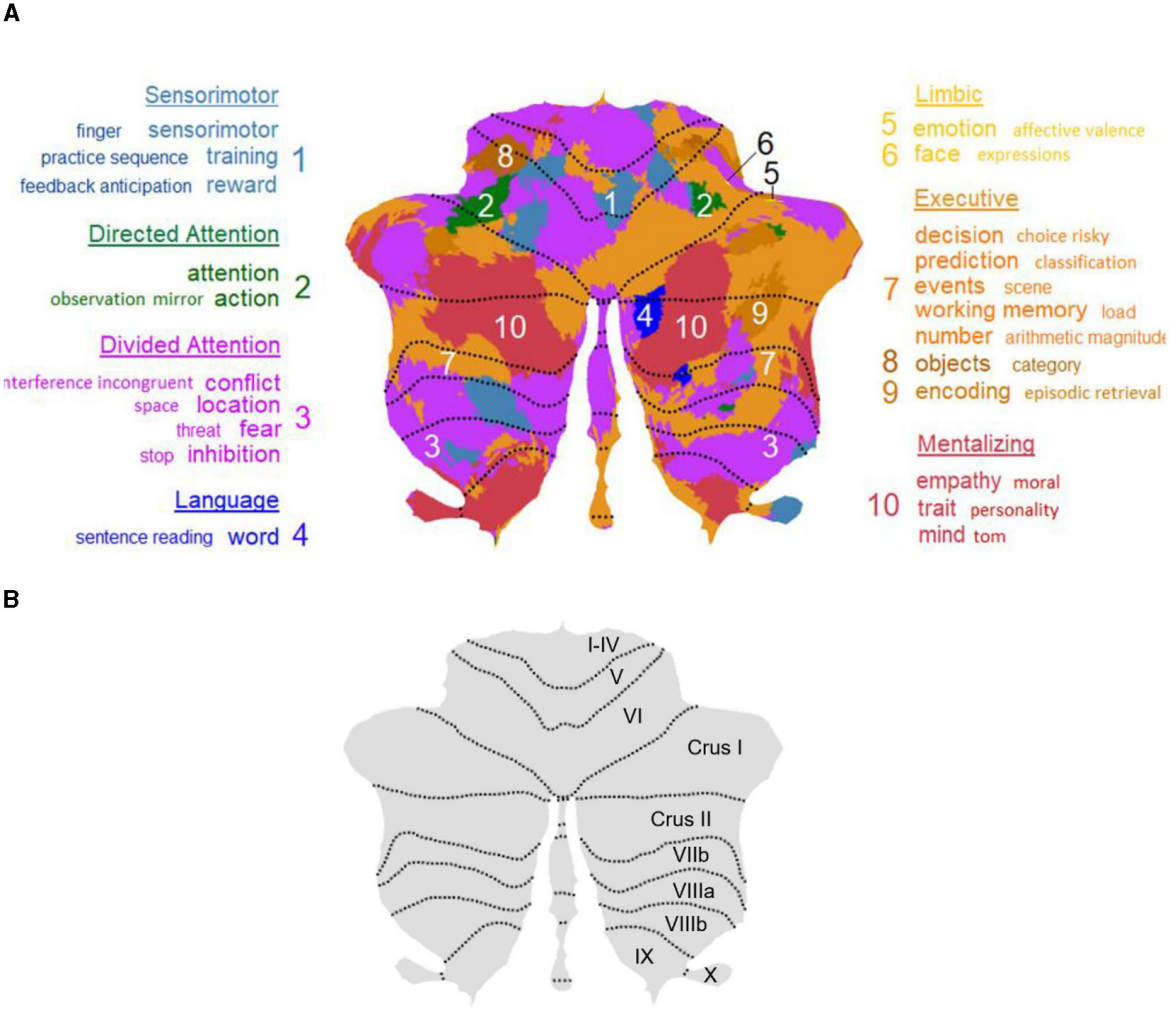
Parcellations of the cerebellum shown by cerebellar flatmaps. **(A)** The 10-cluster task-related cerebellar parcellation based on ALE results from over 1000 fMRI studies from NeuroSynth, suggesting that different functions activate different cerebellar regions (Van Overwalle et al., 2023). The posterior cerebellar Crus I and II and lobule IX supports various mentalizing, language and emotional processes. **(B)** The Cerebellar flat map Atlas.

Social processes are quite complex, and we often use language and emotions to understand, make inferences and communicate in social spaces with other social beings, which might make it difficult to accurately disentangle these processes in practice. Therefore, the aim of the topic is to bring together findings on the cerebellum's role in social, affective, and language processing, which can provide a more general idea about how the cerebellum contributes to higher-level processing.

Coemans et al. explored the effects of anodal cerebellar transcranial direct current stimulation (ctDCS) coupled with language therapy in a bilingual patient with chronic post-stroke aphasia caused by left frontal ischemia. They found significant improvements in untrained repetition and picture description tasks in two languages after anodal ctDCS. Additionally, anodal ctDCS led to improved performance on a cognitive control task, suggesting domain-general enhancements in monitoring abilities. Although these are preliminary results, the study opens promising avenues for further research on ctDCS in aphasia and the role of the cerebellum in language processing.

The study by Niu et al. aimed to investigate the neural mechanisms underlying chronic smoking by analyzing spontaneous brain activity and functional connectivity in smokers. The results showed weakened functional connectivity between the prefrontal cortex and cerebellar subregions. This disrupted connectivity may represent a key role of the cerebellum in emotional regulation, attention, and decision-making associated with chronic smoking and addiction.

Dadomo et al. used multivariate pattern analysis to explore the structural neural correlates of borderline personality disorder, a complex mental disorder characterized by unstable emotions, impulsiveness, feelings of inadequacy, and interpersonal problems. The results showed that morphometric differences in the caudate, putamen and amygdala were predictive of sexual trauma, specifically in this population. The results also showed an association between alterations in cerebellar areas and interpersonal problems in patients with borderline personality disorders.

Finally, the study by Jia et al. investigated the neural basis of schizophrenia by examining functional connectivity in the brain using magnetoencephalography imaging. Reduced social skills and positive symptoms (i.e., hallucinations, delusions or repetitive movements) are key symptoms of schizophrenia. The findings showed that, compared to healthy participants, patients with schizophrenia had distorted functional connectivity across delta-theta, alpha, and beta frequency bands. Specifically, there was increased connectivity in beta frequencies between the left primary auditory cortex and cerebellum, linked to more severe hallucinations in schizophrenia. These findings were interpreted in line with the idea that aberrant agency experience in schizophrenia is the consequence of dysfunctional predictive mechanisms, in turn more broadly in keeping with the view that cerebellar predictive processes are crucially involved in diverse forms of psychopathology (Van Overwalle et al., 2021).

Overall, these studies showed the multifaceted involvement of the cerebellum in a number of high-level processes. Considering the growing consensus that the cerebellum plays a critical role in non-motor domains, it is important to further investigate its contributions. Also, our collected articles showed distorted connectivity between the cerebellum and the corresponding cerebrum in various pathologies, especially those featuring social impairments. Although there is an emerging consensus that cerebellar dysfunction relates to psychiatric disorders, further research is needed to understand this relationship. As a potential target area for brain stimulation, understanding cerebellar functions and its intricate internal and cerebral connections can potentially pave the way for innovative treatments and interventions for clinical conditions related to social processing, emotions and language.

## Author contributions

QM: Writing—original draft. KB: Writing—review & editing. CC: Writing—review & editing. EH: Writing—review & editing.
